# Laboratory-based management of microbiological alerts: effects of an automated system on the surveillance and treatment of nosocomial infections in an oncology hospital

**DOI:** 10.3332/ecancer.2009.137

**Published:** 2009-03-31

**Authors:** R Passerini, R Biffi, D Riggio, S Pozzi, MT Sandri

**Affiliations:** 1 Laboratory Medicine Unit, European Institute of Oncology, Via Ripamonti 435, 20141, Milano, Italy; 2 Abdomino-Pelvic Surgery Division, European Institute of Oncology, Milan, Italy

## Abstract

**Background:**

Prevention and surveillance programs are key to contain Nosocomial Infections (Nis). At the European Institute of Oncology, surveillance based on ***ex-post*** data collection has been done since the inception of hospital activity; laboratory-based surveillance of microbiological alert was not standardized. This study describes the issues related to the recent introduction into the hospital routine of a laboratory-based automated surveillance system and its clinical impact on monitoring and treatment of Nis.

**Methods:**

An interdisciplinary team defined the alerts and the actions to be taken in response; recipients of the alert messages were identified and software was programmed. Program features were created so their employment would generate a prompt notification of clinically critical results. After a training period, the program was introduced in the hospital routine.

**Results:**

There were a total of 150 generated alerts; the main alert related to microorganisms requiring prompt patient isolation and/or public notification. Clinical use of the program was relevant in detection and immediate notification of ***Cytomegalovirus*** active infection in stem cell recipients and central venous catheter related candidemia: the prompt administration of adequate treatment was possible hours earlier compared to the previous approach.

**Conclusions:**

A laboratory-based automated surveillance system is effective in facilitating the management of Nis; its clinical employment also leads to important clinical advantages in patient care.

## Introduction

Nosocomial Infections (Nis) represent a great challenge for hospital personnel as a significant cause of increased mortality and morbidity in hospitalized patients, as well as increased costs of health-care systems; such that their rate is a recognized indicator of quality of care and safety [1,2]. Social-economic and human impact of Nis can be limited by implementing strict prevention actions and active surveillance programs [3]. Appropriate staff education, close adherence to guidelines and established procedures, in addition to continuous training, are the foundation of all effective preventive programs [4,5].Currently, surveillance programs are based on the monitoring of clinically relevant infections through regular data collection and analysis, which allows the evaluation of local incidence of infections and their trends in comparison with retrospective data, as well as to identify outbreaks and risk factors and finally, to compare one’s rates with the literature and/or other institutions’ data. In addition to this periodical monitoring through time, a continuous and timely laboratory-based surveillance assures a prompt identification of sentinel events according to standard requirements [6–8]. The surveillance activity traditionally is performed through the manual review of computerized reports; this methodology however is labour-intensive and neither assures a sensitive detection of sentinel events/outbreaks nor a rapid intervention by infection control professionals; in fact, only the use of an automated system for Nis control, can provide an accurate and real-time surveillance at a lower cost [9,10]. Over the last few years, various electronic Nis surveillance systems have been developed which extract data from the database of microbiology laboratory, clinical wards, pharmacy and admission-transfer-discharge and identify abnormal events and/or microorganism distribution compared to the standard procedure.

The aim of this article was to describe the issues regarding the implementation of a laboratory-based automated system, recently introduced in the hospital routine, which was able to identify microbiological alerts and to notify in real-time the involved professionals. We also analyse its clinical impact on the surveillance and treatment of Nis.

## Methods

This study was prospectively carried out at the European Institute of Oncology (IEO) in Milan, a 226 inpatient-bed comprehensive cancer centre, operating since 1994 in cancer prevention, diagnosis, research and treatment. It includes two medical divisions (Medical Oncology and Haemato-Oncology), eight divisions of oncological surgery and a four-bed Intensive Care Unit (ICU).

At the time we implemented the automated system, the surveillance programs of major Nis had been continuously run at IEO by the application of standardized criteria, addressing: (i) category of infection to be monitored (ventilator-associated pneumonia, nosocomial pneumonia, central line-associated bloodstream infections, surgical site infections of selected surgical procedures and catheter-related urinary tract infections); (ii) data collection frequency (once a month); (iii) data collection sources (from microbiology laboratory reports, surgical wards and operating room databases, pharmacy and clinical records); (iv) data analysis and emission of reports by ICC (Infections Control Committee).

Our objective was to introduce a standardized method for microbiological alert surveillance and notification; thus in 2006, a computerized system for the identification of predefined sentinel events and their notification to clinicians, Chief Medical Office (CMO) and ICC members was implemented and introduced in the hospital routine.

The alert system developed initially involved the Microbiology Laboratory, Abdomino-Pelvic Surgery Division (APSD) and ICU. The software Virtuoso Plus™ (Metafora Informatica Srl, Milano, Italy) has been used, which was able to extract the data directly from the laboratory software and to elaborate the findings.

The early stage of the implementation of the new system was to identify sentinel events. An alert list, based on national and international scientific societies guidelines and recommendations [11], was determined by an interdisciplinary team who integrated these suggestions with IEO specific demands; thus in addition to universally accepted alerts (Methicillin–resistant Staphylococcus aureus (MRSA), Vancomycin-resistant Enterococci (VRE), multi-drug resistant ***P. aeruginosa,*** etc), we added some events critical in our Institute, among which were, for example, ***Aspergillus*** species found in respiratory tract samples (IEO was in the progress of building renovation) [12–14]. To satisfy Nis control, according to the Centers for Diseases Control guidelines, ISS (Istituto Superiore di Sanità, corresponding to NIH-USA) and Regione Lombardia recommendations and IEO specific procedures, a list of actions to be taken in response to each event was issued, including what to do to contain the risk of transmission and how to proceed to notify the CMO and the local Healthcare Authority, when needed (Table 1).

Chief of Microbiology Laboratory, Epidemiology Nurse, Directors and Chief Nurses of the involved Divisions, CMO and ICC Operating Group were identified as recipients of the alert messages. The software setting proceeded: for each identified alert the extraction criteria were defined, including the strain of bacteria isolated, type of clinical sample, patients and divisions involved, recipients of messages and extraction time planning (Table 2).

A standardized alert message, containing a description of the alert event, patient’s data, division of admission, sample examined and microbiological results, has been created to be automatically sent by email to the above recipients. At the same time, a list of all extracted alerts in the current session, with the indication of admission ward, was automatically generated and stored in a directory on the central server, available for evaluation by ICC members: this report allows the location of the contemporary presence of the same event in different patients, thus providing a tool to search for a common infection source (e.g. the same operating room or ICU stay). The extraction of alerts was planned once a day for the routine tests and every two hours for the tests needed urgently.

Due to the possibility of personalization, we resolved to also use this software for a prompt notification to the clinicians of clinically critical results, such as the growth of ***S. aureus*** or ***Candida*** species in blood cultures or ***Cytomegalovirus*** and/or ***Herpes viruses*** genome detection in clinical samples after hematopoietic stem cell transplantation [15–17] among the others; we entered these alerts identifying, however, only the clinicians as message recipient.

After a ten-month training period, involving only APSD and ICU, specifically dedicated to software validation and evaluation of technical functionality, we obtained a positive feedback of its employment and we decided to introduce this surveillance method in the hospital routine. To ensure its correct use by all operators, a new institutional procedure entitled ‘Management of the Microbiological Alerts’ was issued, and the program with its clinical relevance was presented to clinical directors and chief nurses (who in turn showed it to all the nursing staff); some posters to affix in each division, listing alerts and actions to be performed, were also distributed.

## Results

From May 2006 to September 2008, 150 alert notifications were generated from samples sent to the laboratory for microbiological analysis. Table 3 shows these alerts with pertinent data and actions taken.

Multi-drug resistant ***P. aeruginosa***, ***S. maltophilia***, extended-spectrum beta-lactamase (ESBL) and MRSA claimed the infected patients to be promptly placed in contact isolation in order to reduce the risk of direct or indirect transmission [18]. In particular, from January 2008 a significant increase of MRSA was detected, so, in addition to the isolation of patients, we planned some refresher courses on the isolation procedures for professionals, and a survey of our patients colonization at the time of admission and discharge. ***Mycobacteria*** and ***Salmonella*** related alerts led to both the isolation of patients and to public notification. With regard to the clinical employment of the program, ***C. albicans*** strains were isolated from a central and a peripheral blood culture in two patients, with a Differential Time to Positivity [19,20] proving a catheter-related bloodstream infection, thus forcing clinicians to immediately remove the device. The growths of ***S. aureus*** in blood culture were not CVC-related. CMV genome found in three stem cell recipient patients led clinicians to start or change the antiviral therapy.

## Discussion

At the IEO, since the beginning of hospital activity, an Infections Control Committee has ensured Nis surveillance through the review and approval of surveillance and prevention programs, the analysis of Nis collected data and identification of interventional areas, the assessment and promotion of better practices and staff training in infection control, the infectious risk assessment of new technologies and finally the communication and cooperation with external ICC for audit activities.

A rapid detection of microbiological alerts is also crucial for effective control of nosocomial outbreaks, of both common and unusual micro-organisms, and micro-organisms with unusual phenotypical traits (such as multi-drug resistance). Detection of these outbreaks by clinical evaluation is difficult, so it is usually found on periodic reports *ex-post* laboratory collected data, which is a fairly slow method and is late in outbreak detection.

A laboratory-based management of Nis control, on the contrary, allows the rapid survey of alerts; the electronic notification (by email) to the involved divisions ensures, on the other hand, a real-time receipt of the alert messages, thus facilitating the prompt accomplishment of actions required to find the cause, and the immediate implementation of effective measures to contain the risk and to treat infected patients. This was all accomplished following procedures and guidelines previously shared by all the staff. Only the use of an automated system made this surveillance method possible.

In the past, the survey of microbiological alerts at IEO was in the care of Microbiology Laboratory staff, without a standardized method; so, the implementation of an automated surveillance system is shown as a very positive choice, both for the standardization of alert extraction criteria and for the well-timed data reporting to clinicians, with evident advantages: in the case of infectious agents requiring the isolation of patients, for example, the reduction in time of alerts communication reduced the risk of patient-to-patient transmission of microorganisms. The immediate effect is a clinical advantage for the patients and the possibility for the hospital staff to monitor longitudinally the infectious epidemiological setting. The real-time communication of unusual data to division professionals and ICC members, moreover, ensures their continuous knowledge of the Nis current status. In the instances of infections requiring a public notification, this surveillance system allows an automated and documented—by email—notification of relevant data as requested by the Italian Health Care System, including patient anagraphical data, the isolated microorganisms, the date of the sampling and of the report, and finally the ward of admission.

In our experience, major advantages of this software are related to the capability to personalize the extraction criteria: with careful preliminary selection work, both the alerts and the notification’s message recipients can be modified from time to time, according to the hospital needs. A major feature is the possibility of providing a message relevant for clinical use, allowing an immediate implementation of all needed therapeutic actions and a real-time feedback of pertinent information in doubtful cases, so leading to important benefits in patient care: in the CVC-related candidemia, for example, clinicians, promptly alerted, could remove the device and start the correct therapy hours in advance compared to the previous approach, thus decreasing the risk of severe complications connected to this infection [21]; also the immediate signalling to clinicians of CMV genome detection in stem cell recipients allowed the adjustment of treatment without further delay [22].

Finally, the implementation of this system can lead to further results over time, not less significant than the clinical benefits already detected, but related to the educational value of this approach: regular and prompt notification of critical issues to all health-care staff will enhance their awareness for clinically relevant Nis, thus improving a safe environment.

## Figures and Tables

**Table 1:**
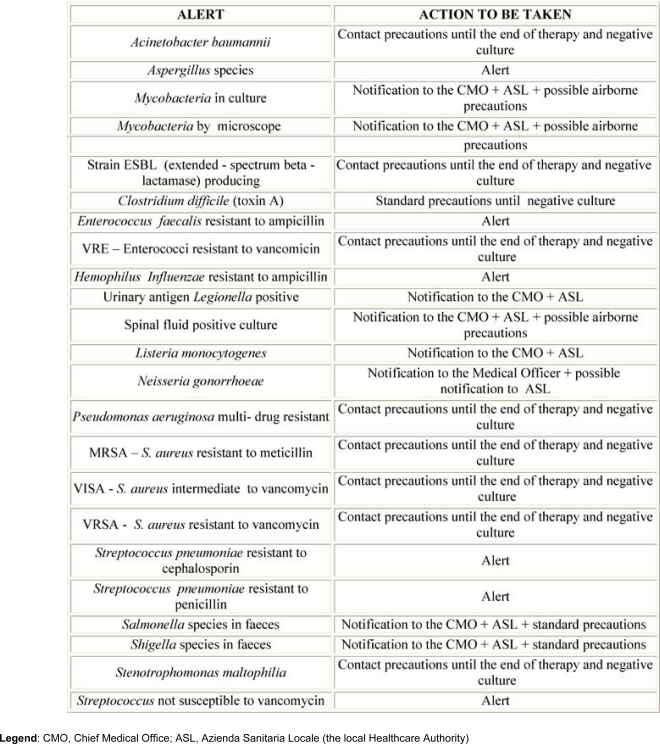
List of alerts and actions to be taken

**Table 2:**
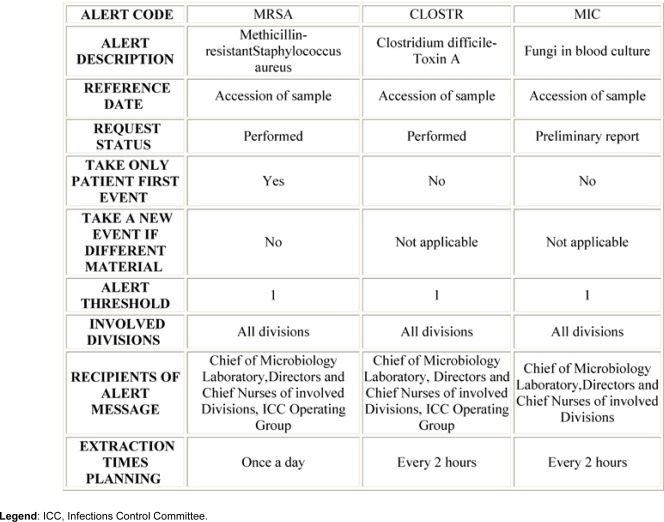
Example of alerts extraction criteria with the software Virtuoso Plus

**Table 3:**
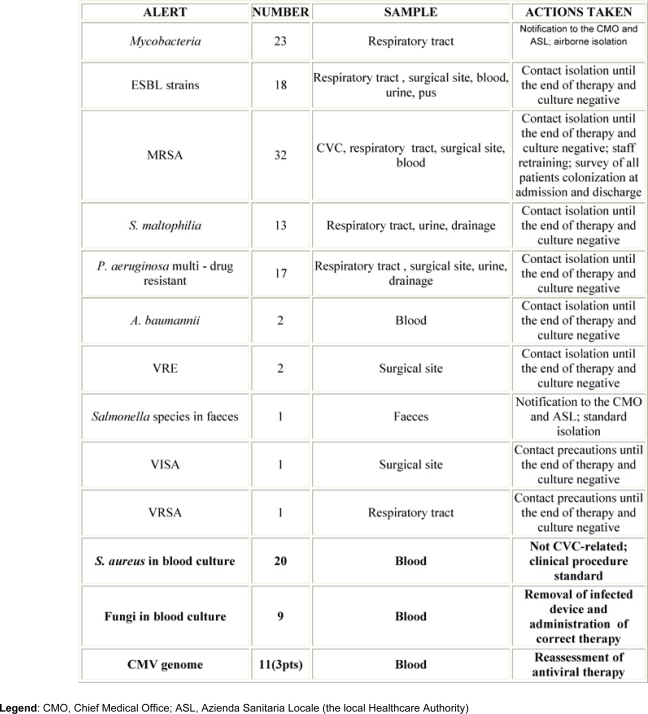
Number of generated alerts and related clinical samples; in bold, clinically critical events
